# Effectiveness of Monovalent and Bivalent mRNA Vaccines in Preventing COVID-19–Associated Emergency Department and Urgent Care Encounters Among Children Aged 6 Months–5 Years — VISION Network, United States, July 2022–June 2023

**DOI:** 10.15585/mmwr.mm7233a2

**Published:** 2023-08-18

**Authors:** Ruth Link-Gelles, Allison Avrich Ciesla, Elizabeth A.K. Rowley, Nicola P. Klein, Allison L. Naleway, Amanda B. Payne, Anupam Kharbanda, Karthik Natarajan, Malini B. DeSilva, Kristin Dascomb, Stephanie A. Irving, Ousseny Zerbo, Sarah E. Reese, Ryan E. Wiegand, Morgan Najdowski, Toan C. Ong, Suchitra Rao, Melissa S. Stockwell, Ashley Stephens, Kristin Goddard, Yessica C. Martinez, Zachary A. Weber, Bruce Fireman, John Hansen, Julius Timbol, Shaun J. Grannis, Michelle A. Barron, Peter J. Embi, Sarah W. Ball, Manjusha Gaglani, Nancy Grisel, Julie Arndorfer, Mark W. Tenforde, Katherine E. Fleming-Dutra

**Affiliations:** ^1^Coronavirus and Other Respiratory Viruses Division, National Center for Immunization and Respiratory Diseases, CDC; ^2^Eagle Health Analytics, San Antonio, Texas; ^3^Westat, Rockville, Maryland; ^4^Kaiser Permanente Vaccine Study Center, Kaiser Permanente Northern California Division of Research, Oakland, California; ^5^Kaiser Permanente Center for Health Research, Portland, Oregon; ^6^Children’s Minnesota, Minneapolis, Minnesota; ^7^Department of Biomedical Informatics, Columbia University Irving Medical Center, New York, New York; ^8^New York-Presbyterian Hospital, New York, New York; ^9^HealthPartners Institute, Minneapolis, Minnesota; ^10^Division of Infectious Diseases and Clinical Epidemiology, Intermountain Healthcare, Salt Lake City, Utah; ^11^School of Medicine, University of Colorado Anschutz Medical Campus, Aurora, Colorado; ^12^Division of Child and Adolescent Health, Department of Pediatrics, Columbia University Vagelos College of Physicians and Surgeons, New York, New York; ^13^Department of Population and Family Health, Columbia University Mailman School of Public Health, New York, New York; ^14^Center for Biomedical Informatics, Regenstrief Institute, Indianapolis, Indiana; ^15^School of Medicine, Indiana University, Indianapolis, Indiana; ^16^Vanderbilt University Medical Center, Nashville, Tennessee; ^17^Section of Pediatric Infectious Diseases, Department of Pediatrics, Baylor Scott & White Health, Temple, Texas; ^18^Department of Medical Education, Texas A&M University College of Medicine, Temple, Texas; ^19^Influenza Division, National Center for Immunization and Respiratory Diseases, CDC.

**Summary**

**What is already known about this topic?**

The original monovalent COVID-19 mRNA vaccines were first recommended in the United States in June 2022 for young children; bivalent vaccines were recommended in December 2022. Postauthorization vaccine effectiveness data in this age group are limited.

**What is added by this report?**

Monovalent and bivalent mRNA vaccines helped provide protection against COVID-19–associated emergency department and urgent care visits among children aged 6 months–4 years (Pfizer-BioNTech) and 6 months–5 years (Moderna).

**What are the implications for public health practice?**

All children should stay up to date with recommended COVID-19 vaccines, including initiating COVID-19 vaccination immediately when they become eligible.

## Abstract

On June 19, 2022, the original monovalent mRNA COVID-19 vaccines were approved as a primary series for children aged 6 months–4 years (Pfizer-BioNTech) and 6 months–5 years (Moderna) based on safety, immunobridging, and limited efficacy data from clinical trials. On December 9, 2022, CDC expanded recommendations for use of updated bivalent vaccines to children aged ≥6 months. mRNA COVID-19 vaccine effectiveness (VE) against emergency department or urgent care (ED/UC) encounters was evaluated within the VISION Network during July 4, 2022–June 17, 2023, among children with COVID-19–like illness aged 6 months–5 years. Among children aged 6 months–5 years who received molecular SARS-CoV-2 testing during August 1, 2022–June 17, 2023, VE of 2 monovalent Moderna doses against ED/UC encounters was 29% (95% CI = 12%–42%) ≥14 days after dose 2 (median = 100 days after dose 2; IQR = 63–155 days). Among children aged 6 months–4 years with a COVID-19–like illness who received molecular testing during September 19, 2022–June 17, 2023, VE of 3 monovalent Pfizer-BioNTech doses was 43% (95% CI = 17%–61%) ≥14 days after dose 3 (median = 75 days after dose 3; IQR = 40–139 days). Effectiveness of ≥1 bivalent dose, comparing children with at least a complete primary series and ≥1 bivalent dose to unvaccinated children, irrespective of vaccine manufacturer, was 80% (95% CI = 42%–96%) among children aged 6 months–5 years a median of 58 days (IQR = 32–83 days) after the dose. All children should stay up to date with recommended COVID-19 vaccines, including initiation of COVID-19 vaccination immediately when they are eligible.

## Introduction

As of June 2023, SARS-CoV-2 had resulted in more than 2 million COVID-19 cases, more than 20,000 hospitalizations, and more than 400 deaths among U.S. children aged 6 months–4 years (*1*,*2*). The original monovalent mRNA vaccines were authorized in June 2022 for children aged 6 months–4 years (Pfizer-BioNTech*) and 6 months–5 years (Moderna^†^) based on safety, immunobridging, and limited efficacy data from clinical trials, with recommendations expanded to include bivalent vaccines in December 2022 (*3*–*5*). Because efficacy data were limited, postauthorization vaccine effectiveness (VE) data are necessary to understand how well the vaccines work and to help guide development of future vaccine policy for this age group.

## Methods

VISION,^§^ a multisite, electronic health care record–based network, evaluated VE against COVID-19–associated emergency department or urgent care (ED/UC) encounters, across six sites in eight states. VISION VE methods have been previously described (*6*). VISION assessed VE among immunocompetent (*7*) children aged 6 months–4 years (monovalent Pfizer-BioNTech, 3-dose primary series) and 6 months–5 years (monovalent Moderna, 2-dose primary series) who visited a participating ED/UC during July 4, 2022–June 17, 2023, with a COVID-19–like illness^¶^ and who received SARS-CoV-2 nucleic acid amplification testing during the 14 days preceding, or up to 72 hours after, the ED/UC encounter. Patients were classified on the index date** as unvaccinated (no COVID-19 vaccine doses received), vaccinated with 1 or 2 monovalent Moderna doses or 1, 2, or 3 monovalent Pfizer-BioNTech doses, or vaccinated with ≥1 bivalent dose. ED/UC encounters were excluded if the most recent vaccine dose was received <14 days before the index date, if the child had received a combination of Moderna and Pfizer-BioNTech vaccine doses, or if a vaccination schedule that was not authorized in the study population had been used (e.g., 4 monovalent Pfizer-BioNTech doses or 3 monovalent Moderna doses). Children who had received bivalent doses were only included if they had a complete primary series (either monovalent or bivalent doses).

VE, stratified by vaccine product and number of doses received, was estimated using a test-negative case-control study design, comparing odds of COVID-19 vaccination versus being unvaccinated in case-patients (those who received a positive SARS-CoV-2 test result) and control-patients (those who received a negative test result).^††^ Analysis periods varied for each product and dose combination based on differences in recommended schedules for Moderna and Pfizer-BioNTech vaccines.^§§^ Children became eligible for inclusion in each analysis 2 weeks after the initial date a child could have received each product and dose combination. Analyses were conducted using R software (version 4.2.2; R Foundation). This study was reviewed and approved by institutional review boards at participating sites or under a reliance agreement with the Institutional Review Board of Westat and was conducted consistent with applicable federal law and CDC policy.^¶¶^

## Results

The 90,905 ED/UC encounters in children aged 6 months–5 years eligible for inclusion in the Moderna monovalent analysis included 4,934 (5.4%) case-patients and 85,971 (94.6%) control-patients (Table 1). An additional 96 encounters occurred among control-patients who received ≥1 bivalent Moderna dose. The 81,077 ED/UC encounters in children aged 6 months–4 years eligible for inclusion in the Pfizer-BioNTech monovalent analysis included 4,642 (5.7%) case-patients and 76,435 (94.3%) control-patients. An additional 222 encounters occurred among children who received ≥1 bivalent Pfizer-BioNTech dose; 219 of these were control-patients, and three were case-patients. 

**TABLE 1:** Characteristics of emergency department and urgent care visits among children aged 6 months–5 years with COVID-19–like illness, by SARS-CoV-2 test result — eight U.S. states, July 4, 2022–June 17, 2023

Characteristic	SARS-CoV-2 test result, no. (column %)			
	Moderna analyses*		Pfizer analyses^†^	
	Positive (case-patients)	Negative (control-patients)	Positive (case-patients)	Negative (control-patients)
**All ED/UC encounters (row %)**	**4,934 (5.4)**	**86,067 (94.6)**	**4,645 (5.7)**	**76,654 (94.3)**
**Variant-predominant period** ^§^				
BA.4/BA.5-related	4,022 (81.5)	63,518 (73.8)	3,756 (80.9)	56,526 (73.7)
XBB-related	912 (18.5)	22,549 (26.2)	889 (19.1)	20,128 (26.3)
**Site**				
Columbia University	376 (7.6)	10,240 (11.9)	346 (7.4)	9,037 (11.8)
HealthPartners and Children’s Minnesota	801 (16.2)	14,196 (16.5)	763 (16.4)	13,197 (17.2)
Intermountain Healthcare	1,825 (37.0)	24,046 (27.9)	1,737 (37.4)	21,718 (28.3)
KPNW	250 (5.1)	4,584 (5.3)	237 (5.1)	3,949 (5.2)
KPNC	1,212 (24.6)	23,629 (27.5)	1,133 (24.4)	20,665 (27.0)
University of Colorado	470 (9.5)	9,372 (10.9)	429 (9.2)	8,088 (10.6)
**Age**				
6–12 mos	1,531 (31.0)	11,866 (13.8)	1,536 (33.1)	11,968 (15.6)
1 yr	1,402 (28.4)	20,188 (23.5)	1,416 (30.5)	20,624 (26.9)
2 yrs	732 (14.8)	16,117 (18.7)	741 (16.0)	16,397 (21.4)
3 yrs	510 (10.3)	14,857 (17.3)	516 (11.1)	15,066 (19.7)
4 yrs	431 (8.7)	12,194 (14.2)	436 (9.4)	12,599 (16.4)
5 yrs	328 (6.6)	10,845 (12.6)	NA	NA
**Sex**				
Female	2,203 (44.6)	38,735 (45.0)	2,089 (45.0)	34,297 (44.7)
Male	2,731 (55.4)	47,332 (55.0)	2,556 (55.0)	42,357 (55.3)
**Race and ethnicity** ^¶^				
Black or African American, non-Hispanic	430 (8.7)	9,261 (10.8)	389 (8.4)	8,012 (10.5)
White, non-Hispanic	1,744 (35.3)	30,379 (35.3)	1,633 (35.2)	27,184 (35.5)
Hispanic or Latino	1,647 (33.4)	28,844 (33.5)	1,567 (33.7)	25,529 (33.3)
Other, non-Hispanic	782 (15.8)	12,195 (14.2)	744 (16.0)	11,163 (14.6)
Unknown	331 (6.7)	5,388 (6.3)	312 (6.7)	4,766 (6.2)
Period**				
Jul 4–31, 2022	914 (18.5)	4,145 (4.8)	847 (18.2)	3,784 (4.9)
Aug–Sep 18, 2022	898 (18.2)	8,569 (10.0)	829 (17.8)	7,658 (10.0)
Sep 19–Dec 23, 2022	1,755 (35.6)	42,680 (49.6)	1,638 (35.3)	37,583 (49.0)
Dec 24, 2022–May 3, 2023	1,250 (25.3)	26,359 (30.6)	1,221 (26.3)	23,815 (31.1)
May 4–June 17, 2023	117 (2.4)	4,314 (5.0)	110 (2.4)	3,814 (5.0)
**Medical condition^††^**				
Asthma	124 (2.5)	5,266 (6.1)	102 (2.2)	4,353 (5.7)
Prematurity	11 (0.2)	248 (0.3)	12 (0.3)	227 (0.3)
Chronic lung disease of prematurity	4 (0.1)	125 (0.2)	5 (0.1)	110 (0.2)
**Vaccination status,^§§^ total no. of doses, vaccine**				
Unvaccinated	4,791 (97.1)	81,573 (94.8)	4,469 (96.2)	71,147 (92.8)
1 dose total, MV Moderna	47 (1.0)	968 (1.1)	NA	NA
2 doses total, MV Moderna	96 (1.9)	3,430 (4.0)	NA	NA
2 doses total, BV Moderna	0 (—)	0 (—)	NA	NA
2 doses total, 1 MV Moderna, 1 BV Moderna	0 (—)	2 (—)	NA	NA
3 doses total, 2 MV Moderna, 1 BV Moderna	0 (—)	94 (0.1)	NA	NA
1 dose total, MV Pfizer-BioNTech	NA	NA	75 (1.6)	1,451 (1.9)
2 doses total, MV Pfizer-BioNTech	NA	NA	70 (1.5)	2,437 (3.2)
3 doses total, MV Pfizer-BioNTech	NA	NA	28 (0.6)	1,400 (1.8)
3 doses total, BV Pfizer-BioNTech	NA	NA	0 (—)	0 (—)
3 doses total, 2 MV Pfizer-BioNTech, 1 BV Pfizer-BioNTech	NA	NA	3 (0.1)	205 (0.3)
3 doses total, 1 MV Pfizer-BioNTech, 2 BV Pfizer-BioNTech	NA	NA	0 (—)	2 (—)
4 doses total, 3 MV Pfizer-BioNTech, 1 BV Pfizer-BioNTech	NA	NA	0 (—)	12 (—)
**Received ≥1 bivalent dose, irrespective of manufacturer**	0 (—)	96 (0.1)	3 (0.1)	219 (0.3)

**Abbreviations:** BV = bivalent; ED = emergency department; ICD-10 = *International Classification of Diseases, Tenth Revision*; KPNC = Kaiser Permanente Northern California; KPNW = Kaiser Permanente Northwest; MV = monovalent; NA = not applicable; UC = urgent care; VE = vaccine effectiveness.

* Children who received Pfizer-BioNTech COVID-19 vaccine were excluded from the Moderna VE analyses. Ninety-six Moderna bivalent dose recipients are included in the table but were excluded from the monovalent Moderna-specific primary series analyses.

^†^ Children who received Moderna COVID-19 vaccine were excluded from the Pfizer-BioNTech VE analyses. A total of 226 recipients of Pfizer-BioNTech bivalent doses are included in the table but were excluded from the monovalent Pfizer-BioNTech–specific primary series analyses.

^§^ Variant predominance was defined as the period during which a variant accounted for ≥50% of all sequenced specimens in the U.S. Department of Health and Human Services region where the site is located. XBB-related sublineages predominated at Columbia University beginning December 31, 2022; at Intermountain Healthcare and University of Colorado beginning January 28, 2023; at HealthPartners and Children’s Minnesota and KPNC beginning February 4, 2023; and at KPNW beginning February 11, 2023.

^¶^ Children whose caregiver reported non-Hispanic ethnicity and any of the following for race were classified as other, non-Hispanic: American Indian or Alaska Native, Asian, Native Hawaiian or other Pacific Islander, or other race, or whose caregiver reported not Hispanic with no corresponding race chosen. Children whose caregiver did not report race and ethnicity were classified as unknown.

** Periods were divided based on when children were eligible for inclusion in analyses according to updates to primary series vaccination policy as follows: July 4, 2022 = 14 days after recommendation of the monovalent primary series; August 1, 2022 = first children eligible for inclusion in analyses of second dose of Pfizer-BioNTech or Moderna monovalent vaccine; September 19, 2022 = first children eligible for inclusion in analyses of third dose of Pfizer-BioNTech monovalent vaccine; December 24, 2022 = first children eligible for inclusion in analyses of bivalent vaccines; and May 4 = first children eligible for inclusion after receipt of bivalent primary series doses. https://www.cdc.gov/vaccines/covid-19/clinical-considerations/interim-considerations-us-appendix.html

^††^ Asthma included children with the following ICD-10 discharge code: J45*; prematurity included children with the following ICD-10 discharge code: P07*; chronic lung disease of prematurity included children with the following ICD-10 discharge code: P27.1. Data on chronic lung disease of prematurity were not available from one site.

^§§^ Vaccination status categories are mutually exclusive. Percentages reflect column percentages among analytic sample and because of exclusion criteria do not reflect vaccine coverage in the population of children with ED/UC encounters. Among all control-children aged 6–23 months, 2–4 years, and 5 years (including children aged 5 years who received the Pfizer-BioNTech 10-*μ*g dose), 10.2%, 13.1%, and 13.4% received ≥1 COVID-19 vaccine dose, respectively. Ten children who received a bivalent dose but did not complete a primary series were dropped, including two children who received 1 bivalent Moderna dose only, four children who received 1 monovalent Pfizer-BioNTech and 1 bivalent Pfizer-BioNTech dose, three children who received 1 monovalent Pfizer-BioNTech dose only, and one child who received 2 bivalent Pfizer-BioNTech doses.

To better understand coverage in this population, receipt of monovalent and bivalent doses among all children aged 6 months–5 years, regardless of dose or product received, including children aged 5 years who received a Pfizer-BioNTech dose, was assessed. Among all 5,131 case-patients identified during July 4, 2022–June 17, 2023, a total of 340 (6.6%) had received ≥1 monovalent doses, and three (0.06%) had received ≥1 bivalent dose, regardless of manufacturer. Among all 92,777 control-patients identified during July 4, 2022–June 17, 2023, a total of 11,195 (12.1%) had received ≥1 monovalent dose, and 384 (0.4%) had received ≥1 bivalent dose, irrespective of manufacturer.

VE of a single monovalent Moderna vaccine dose (partial primary series) in children aged 6 months–5 years was 23% ≥14 days after the dose (median = 64 days after the dose), although the 95% CI included the null value (Table 2). VE of 2 monovalent Moderna vaccine doses (complete primary series) in children aged 6 months–5 years was 46% in the 14–59 days after vaccination (median = 38 days). VE of 2 monovalent Moderna vaccine doses was 21% ≥60 days after vaccination (median = 120 days), although the 95% CI included the null value.

**TABLE 2:** Vaccine effectiveness* against laboratory-confirmed COVID-19–associated emergency department and urgent care encounters among children aged 6 months–4 years (Pfizer-BioNTech analyses) and 6 months–5 years (Moderna analyses), by vaccine product, number of doses, and time since last dose — eight U.S. states, July 2022–June 2023

Vaccine product, age group, analysis period,^†^ no. of doses (time since last dose)	Total	Positive SARS-CoV-2 test result, no. (%)	Median interval since last dose, days (IQR)	VE^§^ (95% CI)
**Monovalent Moderna vaccine, aged 6 mos–5 yrs**				
**1-dose VE analysis, Jul 4, 2022–Jun 17, 2023**				
Unvaccinated (Ref)	86,364	4,791 (5.5)	NA	Ref
1 dose only (≥14 days)	1,015	47 (4.6)	64 (29 to 117)	23 (−4 to 43)
**2-dose VE analysis, Aug 1, 2022–Jun 17, 2023**				
Unvaccinated (Ref)	81,373	3,887 (4.8)	NA	Ref
2 doses (≥14 days)	3,526	96 (2.7)	100 (63 to 155)	29 (12 to 42)
2 doses (14–59 days)	806	23 (2.9)	38 (26 to 49)	46 (17 to 64)
2 doses (≥60 days)	2,720	73 (2.7)	120 (89 to 178)	21 (−1 to 38)
**Monovalent Pfizer-BioNTech COVID-19 vaccine, aged 6 mos–4 yrs**				
**1-dose VE analysis, Jul 4, 2022–Jun 17, 2023**				
Unvaccinated (Ref)	75,616	4,469 (5.9)	NA	Ref
1 dose only (≥14 days)	1,526	75 (4.9)	58 (28 to 106)	7 (−18 to 26)
**2-dose VE analysis, Jul 25, 2022–Jun 17, 2023**				
Unvaccinated (Ref)	72,101	3,828 (5.3)	NA	Ref
2 doses only (≥14 days)	2,507	70 (2.8)	67 (40 to 115)	37 (19 to 51)
2 doses (14–59 days)	1,105	32 (2.9)	37 (25 to 47)	46 (22 to 62)
2 doses (≥60 days)	1,402	38 (2.7)	106 (81 to 155)	27 (−2 to 47)
**3-dose VE analysis, Sep 19, 2022–Jun 17, 2023**				
Unvaccinated (Ref)	62,977	2,829 (4.5)	NA	Ref
3 doses only (≥14 days)	1,428	28 (2.0)	75 (40 to 139)	43 (17 to 61)
3 doses (14–59 days)	563	6 (1.1)	35 (25 to 46)	70 (34 to 87)
3 doses (≥60 days)	865	22 (2.5)	124 (86 to 170)	24 (−17 to 51)
**≥1 bivalent vaccine among children who received at least a complete primary series, irrespective of manufacturer, aged 6 mos–5 yrs^¶^**				
**Dec 24, 2022–Jun 17, 2023**				
Unvaccinated (Ref)	30,146	1,328 (4.4)	NA	Ref
≥1 bivalent dose (≥14 days)	318	3 (0.9)	58 (32 to 83)	80 (42 to 96)**

**Abbreviations:** NA = not applicable; Ref = referent group; VE = vaccine effectiveness.

* VE was calculated as (1 – adjusted odds ratio) x 100%, estimated using a test-negative case-control design, adjusted for age, sex, race and ethnicity, geographic region, and calendar time (days since January 1, 2021).

^†^ Different analysis periods were used for each product and dose number because vaccinated children became eligible to be included 14 days after the dose at different times: 1 dose of Moderna and Pfizer-BioNTech on July 4, 2022; 2 doses of Pfizer-BioNTech on July 25, 2022; 2 doses of Moderna on August 1, 2022; 3 doses of Pfizer-BioNTech on September 19, 2022; and bivalent doses on December 24, 2022.

^§^ Some estimates are imprecise, which might be due to a relatively small number of persons in each level of vaccination or case status. This imprecision indicates that the actual VE could be substantially different from the point estimate shown, and estimates should therefore be interpreted with caution. Additional data accrual could increase precision and allow more precise interpretation.

^¶^ Children included in this estimate were either unvaccinated (received zero COVID-19 vaccine doses) or had received ≥1 bivalent vaccine dose from either manufacturer. Among those who received a bivalent vaccine dose, any combination of monovalent and bivalent doses was included, but at a minimum children had to have received 2 Moderna doses or 3 Pfizer-BioNTech doses (i.e., a complete primary series).

** This estimate was calculated using unadjusted exact methods because of the small number of vaccinated case-patients. All three vaccinated case-patients received bivalent Pfizer-BioNTech doses; vaccinated control-patients included those who received both bivalent Moderna (96) and Pfizer-BioNTech (223) doses.

VE of a single monovalent Pfizer-BioNTech dose (partial primary series) in children aged 6 months–4 years was 7% ≥14 days after the dose (median = 58 days), although the 95% CI included the null value. VE of 2 doses (partial primary series) was 46% during the 14–59 days after the second dose (median = 37 days). VE of 2 doses was 27% ≥60 days after vaccination (median = 106 days), although the 95% CI included the null value. VE of 3 doses (complete primary series) was 70% during the 14–59 days after vaccination (median = 35 days). VE of 3 doses was 24% ≥60 days after vaccination (median = 124 days), although the 95% CI included the null value. VE of ≥1 bivalent dose, irrespective of manufacturer or age group in children aged 6 months–5 years with a complete primary series was 80% ≥14 days after receipt of the last dose (median = 58 days).

## Discussion

In this multisite analysis from the VISION Network, complete primary mRNA COVID-19 vaccination helped protect against ED/UC encounters in young children, although protection waned in patterns similar to those seen in older children and adults (*7*,*8*). In this analysis, receipt of ≥1 bivalent vaccine dose, irrespective of the manufacturer, provided 80% protection for children who had received a complete primary series ≥14 days earlier compared with unvaccinated children; however, few children had received a bivalent dose, so the estimate was imprecise. In addition, the median interval since receipt of the bivalent dose was only 58 days, meaning there was little time for waning to be observed.

A single dose (i.e., an incomplete primary series) of either monovalent Moderna or Pfizer-BioNTech did not provide protection. VE of 2 doses of monovalent vaccine ≥14 days after the second dose was 37% among children aged 6 months–4 years (Pfizer-BioNTech) and 29% among those aged 6 months–5 years (Moderna), aligning with previous data showing effectiveness of ≥2 vaccine doses in young children (*9*). Of note, the predominantly circulating SARS-CoV-2 variants had evolved substantially from the strain included in the original monovalent COVID-19 vaccines by the time young children became eligible, highlighting the importance of receiving an updated vaccine.

To date, limited VE data are available for young children. A previous analysis of national pharmacy testing data showed generally similar patterns of VE by number of doses and time since vaccination in children aged 3–5 years, but higher VE than in the current analysis (*9*). This finding might be related to several factors that might have affected the control populations, including differences among children who are tested at pharmacies compared with those who are treated in an ED/UC, different analysis periods leading to different abilities to assess waning of VE, differences in circulating SARS-CoV-2 subvariants between the two analyses, and differences in circulation of other viruses, including respiratory syncytial virus and influenza.

The median interval since receipt of the most recent dose among children who had not completed their primary series was longer than expected based on the recommended dosing intervals: a median of 64 days since Moderna dose 1 compared with the 4–8 weeks recommended between Moderna doses and a median of 58 days since receiving Pfizer-BioNTech dose 1 versus 3–8 weeks recommended between doses 1 and 2; this aligns with available national data showing that approximately 10% of children aged 2–4 years had received ≥1 COVID-19 vaccine dose and only 6.1% had completed the primary series as of May 2023,*** nearly a full year after vaccines were recommended for this age group.

### Limitations

The findings in this report are subject to at least five limitations. First, VE estimates for Moderna and Pfizer-BioNTech are not directly comparable because of different dates of eligibility for completion of the primary series, which might affect product-specific VE estimates. Different rates of SARS-CoV-2 infection in the population and different circulating subvariants during August 1–September 19, 2022 (when VE could only be assessed for a complete Moderna primary series) compared with September 19, 2022–June 17, 2023 (when VE of a complete primary series for both products could be assessed), likely also affects comparability. Second, vaccination coverage among young children, including those in this analysis, is low, and vaccinated children might systematically differ from unvaccinated children (or from those who initiated but did not complete the primary series) in COVID-19 risk or likelihood of seeking care, which could bias VE results. Third, the combination of low vaccination coverage, relatively low SARS-CoV-2 circulation during the study period, and low overall rates of hospitalization in this age group precluded the assessment of VE against more severe outcomes, which is the primary goal of the U.S. COVID-19 vaccination program. In addition, low bivalent vaccination coverage precluded the estimation of product-specific VE. Fourth, this analysis was not able to control for previous infection because of underreporting in the medical record, which might have resulted in biased estimates. By July–August 2022, among children aged 6–11 months, 12–23 months, and 2–4 years, 66%, 74%, and 83%, respectively, had evidence of infection-induced SARS-CoV-2 immunity.^†††^ These findings should therefore be interpreted as the incremental benefit provided by COVID-19 vaccination in a population with a high prevalence of infection-induced immunity. Finally, because these data are from eight states, this analysis might not be representative of the entire U.S. population.

### Implications for Public Health Practice

Complete Moderna or Pfizer-BioNTech primary series vaccination helped protect against COVID-19–associated ED/UC visits in young children. Although bivalent vaccination coverage was low in this group, ≥1 dose of bivalent vaccine also helped provide protection. All children should stay up to date with recommended COVID-19 vaccines, including initiating COVID-19 vaccination immediately when children become eligible.^§§§^

## Footnotes

* Pfizer-BioNTech is recommended as 3 3-*μ*g doses, with ≥3–8 weeks between doses 1 and 2 and ≥8 weeks between doses 2 and 3.

† Moderna is recommended as 2 25-*μ*g doses separated by ≥4–8 weeks.

§ Sites from the CDC-funded VISION Network that contributed data for this analysis were Columbia University (New York), HealthPartners and Children’s Minnesota (Minnesota and Wisconsin), Intermountain Healthcare (Utah), Kaiser Permanente Northern California (California), Kaiser Permanente Northwest (Oregon and Washington), and University of Colorado (Colorado).

¶ Medical events with a discharge code consistent with COVID-19–like illness were included using *International Classification of Diseases,* *Tenth Revision *(ICD-10) discharge codes: COVID-19 pneumonia: J12.81 and J12.82; influenza pneumonia: J09.X1, J10.0, J10.00, J10.01, J10.08, J11.0, J11.00, and J11.08; other viral pneumonia: J12*; bacterial and other pneumonia: J13, J14, J15*, J16*, J17, and J18*; influenza disease: J09*, J10.1, J10.2, J10.8*, J11.1, J11.2, and J11.8*; acute respiratory distress syndrome: J80; asthma acute exacerbation: J45.21, J45.22, J45.31, J45.32, J45.41, J45.42, J45.51, J45.52, J45.901, and J45.902; respiratory failure: J96.0*, J96.2*, and R09.2; other acute lower respiratory tract infections: J20*, J21*, J22, J40, J41*, J42, J43*, J47*, J85, J85.0, J85.1, J85.2, J85.3, and J86*; acute and chronic sinusitis: J01*; acute upper respiratory tract infections: J00*, J02*, J03*, J04*, J05*, and J06*; acute respiratory illness signs and symptoms: R04.2, R05, R05.1, R05.2, R05.4, R05.8, R05.9, R06.00, R06.02, R06.03, R06.1, R06.2, R06.8, R06.81, R06.82, R06.89, R07.1, R09.0*, R09.1, R09.2, R09.3, and R09.8*; acute febrile illness signs and symptoms: R50* and R68.83; viral infection, not otherwise specified: B34.9; cause-unspecified gastroenteritis and colitis, unspecified: A09 and K52.9; thrombosis: I82.210, I82.290, I82.220, I82.4*, I82.6*, I82.A1*, I82.B1*, and I82.C1*; acute myocarditis: I40.0, I40.1, I40.8, and I40.9. All ICD-10 codes with * include all child codes under the specific parent code.

** The index date for each encounter was defined as either the date of collection of a respiratory specimen associated with the most recent positive or negative SARS-CoV-2 test result before the encounter or the date of the encounter (if testing occurred only after the encounter date).

†† VE was calculated as (1 − adjusted odds ratio) x 100%. Odds ratios and 95% CIs were estimated using multivariable logistic regression controlling for age, race and ethnicity, sex, calendar day (days since January 1, 2021), and geographic region. Calendar day was modeled as natural cubic splines. Odds ratios in strata with sparse data were calculated using unadjusted exact methods.

§§ Children became eligible for inclusion 14 days after receiving the dose at different times: 1 dose of Moderna and Pfizer-BioNTech on July 4, 2022; 2 doses of Pfizer-BioNTech on July 25, 2022; 2 doses of Moderna on August 1, 2022; 3 doses of Pfizer-BioNTech on September 19, 2022; and bivalent doses on December 24, 2022.

¶¶ 45 C.F.R. part 46.102(l)(2), 21 C.F.R. part 56; 42 U.S.C. Sect. 241(d); 5 U.S.C. Sect. 552a; 44 U.S.C. Sect. 3501 et seq.

*** https://covid.cdc.gov/covid-data-tracker/#vaccination-demographics-trends (Accessed July 14, 2023).

†††
https://www.cdc.gov/vaccines/acip/meetings/downloads/slides-2023-06-21-23/03-COVID-Jones-508.pdf

§§§ https://www.cdc.gov/vaccines/covid-19/clinical-considerations/covid-19-vaccines-us.html
